# Prophylaxis and Treatment of Internal Diseases

**Published:** 1906-09

**Authors:** 


PROPHYLAXIS AND TREATMENT OF INTERNAL DISEASES. By Frederick Forchheimer, M.D., Professor of Theory and Practice of Medicine and Clinical Medicine, Medical College of Ohio, University of Cincinnati, Cincinnati, Ohio. Cloth, price $5.00, net.

This book, written for the physician and advanced student, is eminently practical and useful, as it deals with two of the most important phases of the practice of medicine, i. e., the prevention and the treatment of disease. The author has had a wide range of personal experience, and has taken great care to present in a clear, concise manner the essential features of conditions being constantly encountered by the physician. The treatment of disease receives much attention and the remedies which have been most serviceable to Dr. Forchheimer are mentioned first, and are followed by other useful methods recommended by accepted authorities. Hydrotherapy, gymnastics, exercises, diet, etc., are given with a view to their use in private practice. The above book should prove exceedingly valuable to the general practitioner.

